# Incidence and Predictors of Access Site Vascular Complications Following Ultrasound-Guided MANTA Closure Deployment

**DOI:** 10.1177/15266028211059446

**Published:** 2021-12-01

**Authors:** Hirokazu Miyashita, Noriaki Moriyama, Mika Laine

**Affiliations:** 1 Department of Cardiology, Heart and Lung Center, Helsinki University and Helsinki University Central Hospital, Helsinki, Finland; 2 Department of Cardiology and Catheterization Laboratories, Shonan Kamakura General Hospital, Kamakura, Japan

**Keywords:** vascular complication, vascular closure devices, aortic stenosis

## Abstract

**Purpose:**

There is no report on the reproducibility of the ultrasound-navigated MANTA deployment (US-MANTA) technique and little is known about predictors for US-MANTA-related vascular complication (VC). This study aimed to assess the incidence and predictors of access-site VC using the US-MANTA technique and report insights of MANTA-related VC from consecutive cases following large-bore arteriotomy.

**Materials and Methods:**

Consecutive patients who underwent transfemoral transcatheter aortic valve replacement with the US-MANTA technique from November 2018 to February 2020 were evaluated. MANTA-related VC was defined as access-site complications leading to major or minor VCs based on Valve Academic Research Consortium-2 criteria.

**Results:**

Among 378 patients, 23 cases (6.1%) of MANTA-related VC (major VC: n=7 [1.9%], minor VC: n=16 [4.2%]) were identified. No significant difference was observed in the incidence of MANTA-related VC over the observational period (first quartile: 5.3%, second: 5.4%, third: 7.4%, and fourth: 6.3%, p>.50). In 7 patients with MANTA-related major VC, 4 (57.1%) of complications resulted from incomplete apposition of the toggle due to anterior wall calcification of the common femoral artery (CFA). Anterior calcification of the CFA determined by computed tomography was identified as an independent predictor of MANTA-related VCs.

**Conclusions:**

The US-MANTA technique sustainably provides a low rate of access-site VCs following large-bore arteriotomy. Incomplete apposition of the toggle due to anterior calcification of the CFA may lead to ongoing vascular and bleeding complications.

## Introduction

Transcatheter aortic valve replacement (TAVR) is an established treatment for severe aortic stenosis.^1,2^ Although the feasibility of TAVR has been proven, inherent complications are related to the procedure.^3^ Major vascular complication (VC) is a significant cause of death after TAVR via the transfemoral (TF) approach. In previous reports, major VC has been associated with a 2- to 3-fold increase in 30-day mortality and a 2-fold increase in 36-month mortality.^4-6^

The current standard for large-bore vascular closure following TF-TAVR is the use of suture-based vascular closure devices (VCDs). Currently, these conventional approaches are being challenged by a new plug-based MANTA VCD (Teleflex, Wayne, PA, USA) consisting of a bioresorbable intra-arterial polymer toggle and an extravascular large collagen plug. Closure of the arteriotomy is achieved using the toggle-collagen sandwich. A detailed description of MANTA VCD has been previously published.^7^ Several single-arm prospective trials with MANTA revealed that 0% to 4.2% of major VCs occur in patients with favorable transfemoral access.^8-10^ However, retrospective studies showed higher incidences of major VCs (up to 11.0%) in unselected patients compared with selected patients.^11-13^ Previously, we reported the efficacy of the ultrasound-navigated MANTA deployment (US-MANTA) technique; it had a significantly lower incidence of major VC than conventional MANTA deployment without the use of ultrasound navigation (1.5% vs 7.4%, p=0.030), according to the propensity-score matching method.^14^ Although a learning curve was not observed with the use of MANTA VCD,^13^ it is not reported on the US-MANTA technique. There is no report on the reproducibility of the US-MANTA technique and insights of US-MANTA-related VC in a larger population. Therefore, this study aimed to assess the incidence and predictors of access-site VC in consecutive patients who underwent US-MANTA deployment following TF-TAVR in a real-world setting and report insights of MANTA-related VC following large-bore arteriotomy.

## Materials and Methods

### Study Design and Population

**Figure 1.**
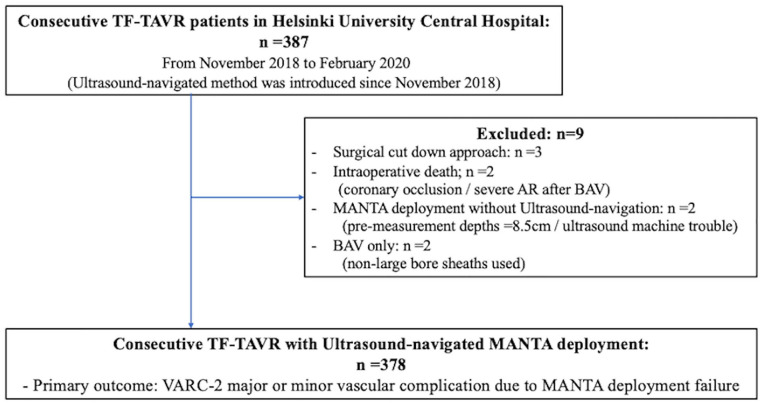
Study flow. The overall population included consecutive 387 TF-TAVR patients. We excluded 9 patients who had surgical cut-down approach (n=3), intraoperative death (n=2) and MANTA deployment without use of ultrasound (n=2) and balloon aortic valvuloplasty (BAV) without use of large-bore sheath (n=2). Three-hundred seventy-eight patients who received ultrasound-navigated MANTA deployment were analyzed. TF-TAVR, transfemoral transcatheter aortic replacement; VARC-2 = Valve Academic Research Consortium-2.

**Figure 2.**
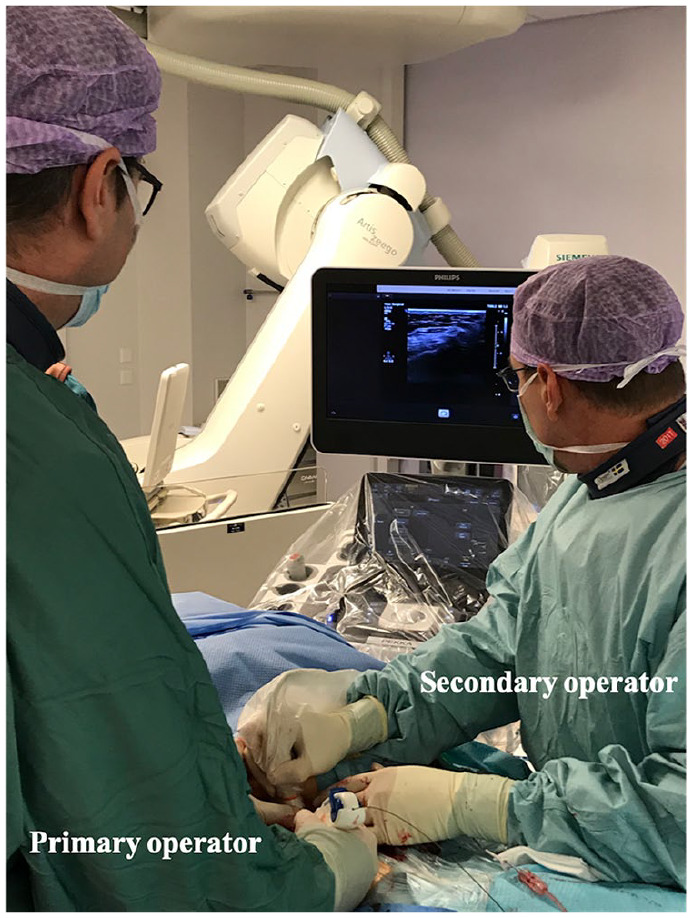
Ultrasound-navigated MANTA method in Helsinki University Hospital. The primary operator stands on the right side of the patient and controls MANTA device under ultrasound image provided by the second operator who stands on left side of that with handling ultrasound probe.

In total, 387 consecutive patients who underwent TF-TAVR between November 2018 and February 2020 at our institution were retrospectively reviewed (Figure 1). All TF-TAVR procedures were planned after contrast-enhanced multi-detector computed tomography (MDCT) and coronary angiography examinations. All patients were evaluated as eligible for TF-TAVR by a multi-disciplinary heart team.^15-17^ The study excluded patients with the surgical cut-down approach, intraoperative death, balloon aortic valvuloplasty with a non-large bore sheath, and conventional MANTA deployment without the use of the ultrasound-navigation method. If eligible patients had previous femoral vascular closure within 30 days^18^ or any previous surgical cut-down, the other side femoral puncture was employed. Activated clotting time was controlled below 250 seconds, and systolic blood pressure was lowered below 120 mmHg at the end of the procedure. An 18-Fr MANTA VCD was applied in all patients who underwent TF-TAVR regardless of the sheath’s outer diameter. The MANTA device has been described in detail previously.^8^ Descriptions of the US-MANTA technique and classification of MANTA deployment failure are summarized in Table 1 and Figure 2, based on our previous report.^14^ Typical and bail-out cases of US-MANTA deployment are shown in Supplementary material, Online Video 1.

**Table 1.** Description of Ultrasound-Navigated MANTA Method and Classification of MANTA Failure.

Technical description of ultrasound-navigated MANTA
Vascular access is established under ultrasound-navigated puncture avoiding anterior wall calcification (avoiding type 3 failure), lateral wall, bifurcation of CFA. Before removing large-bore sheath, pre-shaped stiff wire may be exchanged to straight or small J-tip stiff wire. A scanning in a longitudinal view is used to identify the CFA with MANTA toggle in situ. **Step 1**: An ultrasound image is maintained, and the MANTA dedicated sheath is withdrawn up to pre-determined depth + 1cm. Toggle should be confirmed located in the CFA, then released. 1. If severe posterior calcification is located at the rear part of toggle, sheath should be pulled back further under an ultrasound image. Then, toggle is released in order to avoid toggle stacking due to posterior wall calcification (type 2 failure). 2. If pre-determined deployment depth is not considered reliable, a new deployment depth is visually determined by confirming the toggle locating inside the CFA. **Step 2:** The assembly is pulled back slowly under maintaining an ultrasound image centered on the toggle with 45 degrees or more between skin surface and sheath. The toggle should be confirmed attaching to the anterior vessel wall in parallel to avoid type 1 failure. If toggle stacking due to posterior wall calcification occurred, the assembly should be pushed forward and released from calcification. Then, assembly is pulled back again with a rotating device by 30 to 45 degrees. **Step 3:** Pulling force is maintained under monitoring by the color code of the tension as with the toggle attaching vessel wall in parallel under an ultrasound image until the collagen pad getting close to the arteriotomy. Then, the blue tamper tube is advanced to further compact the collagen pad with keeping pulling force to avoid type 1, 4, and 5 failures at this stage under ultrasound navigation. If collagen delivery failure (type 5 failure) is suspected, the blue tamper tube is further advanced while rotating axially with changing the angle between skin surface and assembly. **Step 4:** Hemostasis is briefly confirmed in visual. Then, the stiff wire should be removed from CFA with keeping the pushing force of the blue tamper. Manual compression with gentle pressure is added if needed.
Classification of MANTA deployment failure
**Type 1:** Toggle protrusion from CFA (and skin surface) **Type 2:** Stuck toggle due to posterior wall calcification **Type 3:** Inappropriate closure due to anterior wall calcification **Type 4:** Aberrant collagen pad inside CFA **Type 5:** Collagen delivery failure

This table was quoted from the previous literature^14^ and updated based on our clinical experience. Abbreviation: CFA = common femoral artery.

### Femoral Vasculature Measurement

For iliofemoral artery assessment, a 3-dimensional MDCT image was retrospectively reconstructed from raw DICOM data using 3mensio Structure Heart software (3mensio Medical Imaging B.V., Bilthoven, The Netherlands). A curved multiplanar reconstruction centerline was generated to assess the cross-sectional image.^19^ The following measurements were obtained in all patients on the side of delivery sheath placement at the level of the common femoral artery (CFA): minimum, mean, and maximum lumen diameter of the vessel at the minimum lumen diameter (MLD) level of the targeted CFA and degree of calcification (defined using visual assessment: 0, no calcification; 1, mild; 2, moderate; and 3, severe).^20^ In addition, the circumferential extent of calcification was assessed in the cross-sectional view at the MLD level. Those whose anterior CFAs were calcified from the 9-o’clock to 3-o’clock position were classified as having anterior calcifications, and vice versa as having posterior calcifications. A CFA eccentricity was calculated as MLD / maximum lumen diameter.^20,21^ The sheath-to-femoral artery ratio (SFAR) was defined as the sheath outer diameter divided by the access-site vasculature MLD.^20,22^ Skin to artery depth was defined as the length between the skin surface to the anterior wall of the vessel at the level of the femoral head in the axial plane of MDCT. The CFA length was defined as the length between the inferior epigastric artery and the ostium of the deep femoral artery.

### Definitions and Outcome Measures

Baseline data, procedural characteristics, and outcomes were collected in a dedicated database. Vascular and bleeding complications were categorized based on the Valve Academic Research Consortium (VARC)-2 definition.^22^ MANTA-related VC was defined as an access-site complication related to the MANTA VCD leading to VARC-2 major or minor VCs. Any complications were observed during the TAVR hospitalization. Access-site complications were evaluated and adjudicated as being related to the MANTA or not by all investigators of this study and vascular surgeons based on US-image and/or surgical inspection at the time of vascular complication.

The primary outcome of this study was the incidence of MANTA-related VCs under ultrasound navigation. The secondary outcomes were the reproducibility of the US-MANTA technique and the independent predictors of US-MANTA deployment failure. In the reproducibility analysis of the US-MANTA technique, 378 patients were divided into 4 groups (group 1=first 95 patients, group 2=second 95 patients, group 3=third 95 patients, and group 4=fourth 93 patients).

### Ethics Statements

Written informed consent was obtained from patients for the regular TF-TAVR procedure. The study protocol conformed to the Declaration of Helsinki and was approved by the institutional review board at our institution.

### Statistical Analysis

Categorical variables are presented as a count and/or percentage and were compared using the chi-square test. Continuous variables are presented as the mean ± standard deviation and were compared using the Student *t* test or Wilcoxon rank sum test based on their distributions. To determine predictors of MANTA-related VC, a logistic regression analysis including baseline, MDCT, and procedural covariates was used to obtain the odds ratio (OR) and 95% confidence interval (CI) for the development of endpoints. Variables with a p value <0.1 in univariate analysis (minimum lumen diameter, eccentricity, SFAR, and anterior calcification) were included in the multivariate model 1. SFAR and anterior calcification were evaluated in the multivariable model 2 which was created to avoid overfitting in model 1. A p value <0.05 was considered statistically significant. All statistical tests were 2-tailed, and statistical analyses were performed using JMP version 10.0 (SAS Institute Inc, Cary, NC, USA).

## Results

### Patient Characteristics and MANTA-Related Vascular Complications

**Figure 3.**
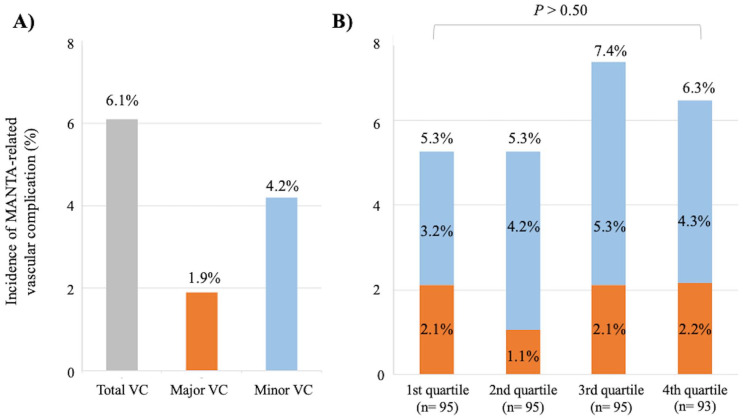
The incidence of MANTA-related vascular complication. In total, 6.1% of MANTA-related vascular complications were identified (major vascular: 1.9%, minor vascular: 4.2%) (A). No significant difference was observed in the incidence of MANTA-related vascular complications across the quartiles (B). VC, vascular complication.

Our analysis included 378 patients who underwent US-MANTA deployment following TF-TAVR (Figure 1). Among those, 23 cases (6.1%) of MANTA-related VC (major VC: n=7 [1.9%], minor VC: n=16 [4.2%]) were identified (Figure 3A). Baseline characteristics and MDCT variables with and without MANTA-related VC are displayed in Table 2. Although there was no significant difference in baseline characteristics between the 2 groups, patients with MANTA-related VC had more frequent anterior calcification (52.2% vs 8.5%, p<0.001) than those without MANTA-related VC. No significant differences were observed in procedural characteristics between the 2 groups (Table 3). Figure 3B shows no significant differences in the incidence of MANTA-related VCs across quartiles.

**Table 2.** Baseline Clinical Characteristics and Preprocedural Computed Tomography Evaluation for Common Femoral Artery.

	All patients n=378	MANTA-VC (-) n=355 (93.9%)	MANTA-VC (+) n=23 (6.1%)	p value
Age, years	80.5±6.5	80.6±6.4	79.3±7.4	0.383
Female	177 (46.8)	116 (46.8)	11 (47.8)	0.921
BMI, kg/m^2^	26.2±4.7	26.1±4.7	26.8±5.4	0.496
BSA, m^2^	1.83±0.20	1.83±0.20	1.82±0.20	0.746
Hypertension	340 (90.0)	318 (89.6)	22 (95.7)	0.3478
Diabetes mellitus	102 (27.0)	94 (26.5)	8 (34.8)	0.3846
CKD	153 (40.6)	144 (40.7)	9 (39.1)	0.884
Atrial fibrillation	143 (37.0)	136 (38.3)	7 (30.4)	0.450
COPD	94 (24.9)	91 (25.6)	3 (13.0)	0.068
Peripheral artery disease	45 (11.9)	40 (11.3)	5 (21.7)	0.133
Prior PCI	95 (25.1)	87 (24.5)	8 (34.8)	0.271
Prior CVA/TIA	29 (7.7)	27 (7.6)	2 (8.7)	0.849
STS-PROM	4.2±1.9	4.2±1.8	4.3±1.9	0.724
Laboratory data				
Hemoglobin, g/L	126.1±15.8	126.3±15.6	124.3±17.7	0.565
Platelet count, 10^3^/mm^3^	213.8±73.8	214.2±74.9	209.4±51.4	0.764
eGFR, mL/min/1.73 m^2^	63.6±18.2	63.6±18.1	63.4±20.0	0.974
Medical therapy				
Single anti-platelet therapy	148 (39.2)	140 (40.5)	8 (34.8)	0.658
Dual anti-platelet therapy	33 (8.7)	30 (8.5)	3 (13.0)	0.450
Oral anti-coagulants therapy	131 (34.8)	123 (34.8)	8 (34.8)	0.990
Vitamin-K antagonist	57 (15.1)	54 (15.2)	3 (13.0)	0.774
DOAC	74 (19.6)	69 (19.4)	5 (21.7)	0.787
CFA variables (CT evaluation)				
Skin to artery depth, mm	34.1±17.3	34.0±17.0	34.3±21.4	0.954
Length of CFA, mm	40.5±12.3	40.2±12.2	44.2±13.9	0.132
High take-off profunda artery	20 (5.3)	19 (5.4)	1 (4.4)	0.835
Mean lumen diameter, mm	8.0±1.2	8.0±1.2	7.8±1.3	0.266
Minimum lumen diameter, mm	7.1±1.3	7.2±1.3	6.7±1.3	0.070
Maximum lumen diameter, mm	8.9±1.4	8.9±1.4	8.8±1.5	0.752
Eccentricity	0.81±0.11	0.81±0.11	0.76±0.12	0.052
SFAR	1.08±0.22	1.08±0.21	1.17±0.31	0.055
Calcification severity				0.335
None to mild	308 (81.5)	291 (82.0)	17 (73.9)	
Moderate to severe	70 (18.5)	64 (18.0)	6 (26.1)	
Angle of calcification, degree	42.2±60.0	41.4±59.3	55.1±68.9	0.287
Anterior calcification	42 (9.8)	30 (8.5)	12 (52.2)	<0.001
Posterior calcification	178 (47.1)	165 (46.5)	13 (56.5)	0.350

Continuous data are presented as the means ± standard deviation; categorical data are given as the counts (percentage). Estimated glomerular filtration rate <60 mL/min/1.73 m^2^.

Abbreviations: BMI, body mass index; BSA, body surface area; CABG, coronary artery bypass graft; CFA, common femoral artery; CKD, chronic kidney disease; COPD, chronic obstructive pulmonary disease; CT, computed tomography; CVA/TIA, cerebrovascular attack/transient ischemic attack; DOAC, direct oral-anticoagulant; eGFR, estimated glomerular filtration rate; PCI, percutaneous coronary intervention; SFAR, sheath-to-femoral artery ratio; STS-PROM, Surgeons Predicted Risk of Mortality.

**Table 3.** Procedure Characteristics.

	All patients n=378	MANTA-VC (-) n= 355 (93.9%)	MANTA-VC (+) n= 23 (6.1%)	p value
Sheath size, inner diameter (Fr)	16.2±2.4	16.1±2.4	16.5±2.7	0.485
Pre-dilatation	276 (73.0)	259 (73.0)	17 (73.9)	0.920
Post-dilatation	27 (7.1)	26 (7.3)	1 (4.4)	0.591
Implanted THV				0.485
SAPIEN 3 / Ultra	145 (38.4)	139 (39.2)	6 (26.1)	
ACURATE neo	110 (29.1)	102 (28.7)	8 (34.8)	
Evolut R / Pro	91 (24.1)	84 (23.7)	7 (30.4)	
Allegra	9 (2.4)	9 (2.5)	0 (0)	
Portico	9 (2.4)	9 (2.5)	0 (0)	
LOTUS Edge	14 (3.7)	12 (3.4)	2 (8.7)	
THV size, mm	27.0±3.6	27.0±3.6	26.7±4.0	0.711
Second valve required	2 (0.5)	2 (0.5)	0 (0)	0.833

Continuous data are presented as the means ± standard deviation; categorical data are given as the counts (percentage).

Abbreviations: CFA, common femoral artery; Fr, French; THV, transcatheter heart valve.

### Clinical Outcomes and Predictors of MANTA Deployment Failure

Clinical outcomes following TF-TAVR are summarized in Table 4. Patients without MANTA-related VC had a significantly lower incidence of total vascular and bleeding complications than those with MANTA-related VC. There were 5 major VCs in patients without MANTA-related VC. Three patients developed left ventricular perforation due to the stiff wire. The other 2 patients had iliac artery rupture and late retroperitoneal hematoma, respectively. In addition, patients without MANTA-related VC had a significantly lower hemoglobin decline (17.0±15.5 vs 26.6±12.8 g/L, p=0.005) and shorter hospital stay (1.3±2.8 vs 3.4±1.8 days, p=0.012) than those with MANTA-related VC did. MANTA-related major VCs are described in detail in Table 5. Of 7 cases of major VCs, 4 complications (57.1%) resulted from anterior wall calcification of the targeted CFAs by ultrasound inspection (type 3 failure). Types 1, 2, 4, and 5 failures were observed in 1 patient each, respectively. Two cases had complex types of failure (types 2+4 and types 3+5). One patient developed a MANTA-related major VC, which was not classified because a stiff wire was stuck inside the MANTA assembly, and the wire could not be removed. Then, we had to remove MANTA and the wire out of the vessel, leading to surgical repairment. There were 16 patients with MANTA-related minor VCs; 9 were hematoma, and 7 were MANTA VCD failure which did not meet major VC criteria. Out of 7 MANTA VCD failures, 2 patients had type1 failure, the other 2 had type 5 failure, the other 1 had type 2 failure, and the other 1 had complex types of 2+4 failure.

**Table 4.** Clinical Outcomes Following Transfemoral Transcatheter Aortic Valve Replacement With MANTA.

	All patients n=378	MANTA-VC (-) n= 355 (93.9%)	MANTA-VC (+) n= 23 (6.1%)	p value
MANTA-related vascular complications				
Major	7 (1.9)	—	7 (30.4)	
Minor	16 (4.2)	—	16 (69.6)	
MANTA-related bleeding complications				
Life-threatening	2 (0.7)	—	2 (8.7)	
Major	5 (1.8)	—	5 (21.7)	
Minor	4 (1.4)	—	4 (17.4)	
Hemoglobin drop (before – after TAVR), g/L	17.5±15.4	17.0±15.5	26.6±12.8	0.005
Total major vascular complication	13 (3.4)	5 (1.5)	8 (34.7)	<0.001
Total minor vascular complication	21 (5.6)	5 (1.5)	16 (69.6)	<0.001
Total life-threatening or major bleeding	14 (3.7)	7 (1.9)	5 (21.7)	<0.001
Total minor bleeding	22 (5.8)	8 (2.3)	14 (60.9)	<0.001
Stroke	6 (1.6)	6 (1.7)	0 (0)	0.530
AKI	2 (0.5)	2 (0.6)	0 (0)	0.718
De novo permanent pacemaker implantation	23 (6.1)	23 (6.5)	0 (0)	0.208
Hospital stay after TAVR, days	1.5±2.7	1.3±2.8	3.4±1.8	0.012
In-hospital mortality	2 (0.5)	2 (0.5)	0 (0)	0.723

Continuous data are presented as the means ± standard deviation; categorical data are given as the counts (percentage).

Abbreviations: AKI, acute kidney injury; TAVR, transcatheter aortic valve replacement; VC, vascular complication.

**Table 5.** Detailed Description of MANTA-Related Major Vascular Complications.

Case of major VC	Failure type	Description
1	1	Overt bleeding with hemoglobin drop >3g/dL, surgical treatment (suturing)
2	3	Pseudoaneurysm requiring surgical treatment (suturing and hematoma removal). Hemoglobin drop >3g/dL.
3	2, 4	Occlusion of CFA after MANTA deployment requiring surgical treatment with 3 units of RBC transfusion. Toggle was stacked on posterior calcification and elevated inside CFA. Part of collagen pad migrating inside vessel was confirmed.
4	3	Overt bleeding with hemoglobin drop >5g/dL with 3 units of RBC transfusion, surgical treatment (suturing)
5	—	Successful MANTA deployment was confirmed under ultrasound image. At the final stage of deployment, SAFARI stiff wire (Boston Scientific, Natick, MA) was stacked inside MANTA assembly. Successfully, it was pulled out with strong force. Few hours later, severe retroperitoneal bleeding requiring surgical treatment with vasopressor injection, 6 units of RBC transfusion, and hemoglobin drop >5g/dL was occurred.
6	3, 5	Overt bleeding with hemoglobin drop >3g/dL requiring surgical treatment (suturing). Collagen delivery failure due to interference by inguinal ligament (type 5). Moreover, type 3 complication was also suspected by ultrasound image.
7	3	Pseudoaneurysm formation requiring surgical treatment with hemoglobin drop >3g/dL. Ultrasound image retrospectively confirmed MANTA toggle stacking due to anterior wall calcification.

Abbreviations: CFA, common femoral artery; RBC, red blood cell, VC, vascular complication.

Predictors of MANTA-related VC are displayed in Table 6. Anterior wall calcification of the CFA (model 1, OR: 3.78, 95% CI: 1.23 to 10.6; model 2, OR:3.96, 95% CI: 1.32 to 10.9) was identified as an independent predictor of MANTA-related VC by multivariate analysis.

**Table 6.** Factors Associated With MANTA Deployment Failure.

Model 1	Univariate			Multivariate		
Variables	OR	(95% CI)	p value	OR	(95% CI)	p value
Minimum lumen diameter	1.37	(0.84, 1.97)	0.06	1.24	(0.58, 2.40)	0.57
Eccentricity	4.61	(1.02, 9.31)	0.05	2.31	(0.06, 30.2)	0.56
SFAR	5.26	(0.89, 27.1)	0.07	1.47	(0.03, 34.5)	0.84
Anterior calcification	4.74	(1.71, 12.1)	0.002	3.78	(1.23, 10.6)	0.02
Model 2						
Variables	OR	(95% CI)	p value	OR	(95% CI)	p value
SFAR	5.26	(0.89, 27.1)	0.07	1.52	(0.16, 33.6)	0.34
Anterior calcification	4.74	(1.71, 12.1)	0.002	3.96	(1.32, 10.9)	0.02

Abbreviations: CI, confidence interval; OR, odds ratio; SFAR, sheath-to-femoral artery ratio.

## Discussion

This is the first study to systematically analyze MANTA-related VCs with the novel US-MANTA deployment technique. The main findings of this study are as follows: (1) MANTA-related VCs occurred in 6.1% of cases (major VCs, 1.9%; minor VCs, 4.2%); (2) the incidence of VCs was sustainably low during the study period (>1 year), and (3) anterior wall calcification of the CFA derived by pre-procedural MDCT measurements was significantly associated with MANTA-related VCs. In addition, this study reported failure mechanisms of US-MANTA deployment that led to VARC-2 major VCs in detail.

### Efficacy of Ultrasound-Navigated MANTA Deployment

Our study described the technique of US-MANTA deployment in detail^14^ and its outcome in consecutive patients who underwent TF-TAVR requiring large-bore arteriotomy. Some reports previously showed the feasibility and safety of the conventional MANTA deployment without US navigation compared with suture-based VCDs in terms of vascular and bleeding complications following TF-TAVR.^11,12,23^ A recent clinical study showed that MANTA VCD provided a low major complication rate (1.9%) in percutaneous endovascular aneurysm repair or thoracic endovascular aortic repair patients.^24^ However, the incidence of major VCs with MANTA still varied from 0% to 11%, which could be depending on the population and procedural variance included in the studies.^8-13,24^ When comparing a suture-based VCD with and without ultrasound following TAVR, the ultrasound-guided technique significantly reduced VC.^25^ In our current and previous studies of the US-MANTA technique,^14^ the rates of major VCs (1.9 and 1.5%, respectively) were in the lower range of those reported in previous studies.^8-14,24^ Also, our previous study demonstrated a propensity score-matched comparison between US-MANTA versus conventional MANTA, which concluded that the US-guided technique was an independent predictor of less frequent access-site major vascular complications.^14^ These results suggest that the ultrasound-guided technique might be beneficial. Furthermore, the US-MANTA technique could be one of the solutions to optimize the use of MANTA and minimize the incidence of VCD-related complications following procedures requiring a large-bore sheath with steady and sufficiently high success rates. However, the present study did not include the data on the number of potential failures that were managed with the US-guided technique. Further studies assessing the efficacy of avoiding the potential failure under US guidance are warranted.

### MANTA-Related Vascular Complication

Although US-MANTA deployment achieved a low incidence of access-related complications, several major VCs were still observed in this study. Although all of the CFAs were punctured under ultrasound imaging using the short-axis view to avoid calcification, anterior wall calcification was found to be an independent predictor of access-related VC in this study. Previous studies of suture-based VCD revealed anterior calcification as the predictor of vascular complication.^26,27^ These findings are reasonable as suture-based VCD works by suturing the arteriotomy, mostly anterior vessel wall. As to MANTA VCD, anterior calcification might cause incomplete sealing and prevent the toggle from sealing the arteriotomy. In our study, although 3 of 4 patients with major VC due to anterior calcification were relatively healthy and had a wide enough targeted puncture site on calcified CFAs according to pre-procedural MDCT, type 3 MANTA deployment failure happened unexpectedly. These findings could imply that performing arterial puncture with short-axis ultrasound imaging is insufficient to avoid anterior calcification. As reported previously, the long-axis approach to vascular access under ultrasound navigation is associated with improved visibility of the needle tip compared with the short-axis approach;^28^ thus, the long-axis approach may have the potential to further reduce VCs related to anterior calcification. With a long-axis scan of the CFA, identifying the distribution of calcification and confirming the needle tip may be essential to establishing large-bore arteriotomy even in the era of plug-based vascular closure. If anterior calcification close to the arteriotomy is identified during US-MANTA deployment even though careful puncture has been done, operators should recognize the risk of percutaneous closure failure requiring additional surgical repair. Until successful vascular closure with MANTA can be performed, contralateral femoral access should be kept for bail-out endovascular therapy, such as balloon occlusion and stent-graft implantation, and surgical repair. Recent studies with conventional MANTA deployment have demonstrated that femoral artery diameter and severe CFA calcification were independent predictors.^29,30^ Univariate analysis in our study revealed the minimum lumen diameter as borderline significance for MANTA-related VCs, while multivariate analysis did not show significance. The small diameter of CFA may have an impact on VCD failure because the small vessel size had limited space to open the toggle appropriately; however, our study did not reveal it as the predictor. One possible explanation might be that the US-guided technique could visualize the right position to open the toggle even in small vessels. Regarding CFA calcification, the question then arises as to the variables and definition of CFA calcification. They were different across the studies,^25-27,29,30^ thus the predictors might be different among them. One of the studies with suture-based VCD defined vessel wall calcification classification as anterior, posterior, lateral, and medial calcification, and found out that only anterior calcification predicted additional VCD deployment.^26^ Lateral and medial wall calcification might be difficult to visualize with a long-axis ultrasound view. Moreover, the operator might puncture lateral or medial wall due to mostly poor ultrasound images, hence lateral or medial calcification might have a negative effect in certain settings with MANTA VCD. Further studies to elucidate optimal puncture, closure techniques, patient selection, and measuring method of CFA calcification are warranted.

### Future Directions

Montero-Cabezas et al^31^ reported the successful use of MANTA for fully percutaneous decannulation of femoral extracorporeal membrane oxygenation (ECMO) cannulation. After ECMO cannulation, the pre-measurement of the depth from the skin to the vessel is considered impossible with the puncture locating dilator of MANTA. However, the ultrasound method enables operators to confirm the toggle position appropriately even without pre-measurement. Therefore, the use of the ultrasound method may be effective for this situation, as reported previously by Dahlbacka et al.^32^ For the same reason, the US-MANTA technique could also be useful as a bail-out method for torrential bleeding post-failure of pre-closure with suture-based VCDs as long as the procedure wire is left within the complicated vessel. Moreover, it is important to investigate when to perform an endovascular therapy instead of surgical repairment, as bail-out in the case of MANTA deployment failure. Further studies and clinical experiences with MANTA and the use of ultrasound navigation are warranted.

### Limitations

First, this was a retrospective single-center study with typical limitations. Also, the comparison between the US-MANTA and conventional MANTA technique has been absent except for a small single-center study.^14^ A larger study is warranted to establish the superiority of the US-MANTA technique. Second, we did not account for the effect of unknown confounding factors other than those included in the multivariate model for the incidence of MANTA-related VCs. Third, we used an 18-Fr MANTA VCD for all of the patients regardless of the sheath outer diameter. Therefore, these data may not support the efficacy of the 14-Fr MANTA VCD. Fourth, in the reproducibility analysis, consistency of patient characteristics over the study period was not available. Fifth, there is a possibility that minor complications not worthy of clinical mention may be underreported because of the lack of systematic post-TAVR assessment with angiography and vascular ultrasonography. Moreover, pre- and post-procedural ultrasonography examinations were not performed regularly. Therefore, the incidence of minor VC might be underestimated in the current study with a retrospective study nature. Finally, the current study only assessed the incidence of in-hospital complications, and length of hospital stay was 1.5±2.7 days. Therefore, we could have missed late vascular complication after discharge.

## Conclusion

The US-MANTA technique sustainably provides a low incidence of VCs following large-bore arteriotomy. Incomplete apposition of the toggle due to anterior calcification may lead to ongoing vascular and bleeding complications. Anterior wall calcification of the CFA needs to be considered to avoid MANTA-related VCs. Accordingly, vascular access should be established with ultrasound in a gingerly manner to avoid anterior wall calcification puncture.

## Supplementary Material

Supplemental Video 1

Supplemental Video 2

## References

[1] Mack MJ Leon MB Thourani VH , et al. Transcatheter aortic-valve replacement with a balloon-expandable valve in low-risk patients. N Engl J Med. 2019;380:1695–1705. 30883058 10.1056/NEJMoa1814052

[2] Popma JJ Deeb GM Yakubov SJ , et al. Transcatheter aortic-valve replacement with a self-expanding valve in low-risk patients. N Engl J Med. 2019;380:1706–1715. 30883053 10.1056/NEJMoa1816885

[3] Beohar N Kirtane AJ Blackstone E , et al. Trends in complications and outcomes of patients undergoing transfemoral transcatheter aortic valve replacement: experience from the PARTNER continued access registry. JACC Cardiovasc Interv. 2016;9:355–363. 26803420 10.1016/j.jcin.2015.10.050

[4] Tchetche D Dumonteil N Sauguet A , et al. Thirty-day outcome and vascular complications after transarterial aortic valve implantation using both Edwards Sapien and Medtronic CoreValve bioprostheses in a mixed population. EuroIntervention. 2010;5(6):659–665. 20142215 10.4244/eijv5i6a109

[5] Généreux P Webb JG Svensson LG , et al. Vascular complications after transcatheter aortic valve replacement: insights from the PARTNER (Placement of AoRTic TraNscathetER Valve) trial. J Am Coll Cardiol. 2012;60:1043–1052. 22883632 10.1016/j.jacc.2012.07.003

[6] Laakso T Moriyama N Raivio P , et al. Impact of major vascular complication access site status on mortality after transfemoral transcatheter aortic valve replacement; results from the FinnValve registry. Circ Rep. 2020;2:182–191. 33693226 10.1253/circrep.CR-20-0007 PMC7921363

[7] Van Gils L De Jaegere PP Roubin G , et al. The MANTA vascular closure device: a novel device for large-bore vessel closure. JACC Cardiovasc Interv. 2016;9:1195–1196. 27282604 10.1016/j.jcin.2016.03.010

[8] Van Mieghem NM Latib A van der Heyden J , et al. Percutaneous plug-based arteriotomy closure device for large-bore access: a multicenter prospective study. JACC Cardiovasc Interv. 2017;10:613–619. 28335899 10.1016/j.jcin.2016.12.277

[9] Wood DA Krajcer Z Sathananthan J , et al. Pivotal clinical study to evaluate the safety and effectiveness of the MANTA percutaneous vascular closure device. Circ Cardiovasc Interv. 2019;12:e007258. 10.1161/CIRCINTERVENTIONS.119.007258 31296082

[10] Halim J Missault L Lycke M , et al. Assessment of the MANTA closure device in transfemoral transcatheter aortic valve replacement: a single-centre observational study. Neth Heart J. 2020;28(12):639–644. 32720122 10.1007/s12471-020-01465-3 PMC7683762

[11] Biancari F Romppanen H Savontaus M , et al. MANTA versus ProGlide vascular closure devices in transfemoral transcatheter aortic valve implantation. Int J Cardiol. 2018;263:29–31. 29681408 10.1016/j.ijcard.2018.04.065

[12] Moriyama N Lindström L Laine M . Propensity-matched comparison of vascular closure devices after transcatheter aortic valve replacement using MANTA versus ProGlide. EuroIntervention. 2019;14:e1558–e1565. 10.4244/EIJ-D-18-00769 30295293

[13] Moccetti F Brinkert M Seelos R , et al. Insights from a multidisciplinary introduction of the MANTA vascular closure device. JACC Cardiovasc Interv. 2019;12:1730–1736. 31488301 10.1016/j.jcin.2019.06.049

[14] Moriyama N Dahlbacka S Vähäsilta T , et al. The efficacy of the ultrasound-navigated MANTA deployment following transfemoral transcatheter aortic valve replacement. JACC Cardiovasc Interv. 2019;12:2564–2566. 31857032 10.1016/j.jcin.2019.09.018

[15] Lauck SB Wood DA Baumbusch J , et al. Vancouver transcatheter aortic valve replacement clinical pathway: minimalist approach, standardized care, and discharge criteria to reduce length of stay. Circ Cardiovasc Qual Outcomes. 2016;9(3):312–321. 27116975 10.1161/CIRCOUTCOMES.115.002541

[16] Babaliaros V Devireddy C Lerakis S , et al. Comparison of transfemoral transcatheter aortic valve replacement performed in the catheterization laboratory (minimalist approach) versus hybrid operating room (standard approach): outcomes and cost analysis. JACC Cardiovasc Interv. 2014;7:898–904. 25086843 10.1016/j.jcin.2014.04.005

[17] Moriyama N Vento A Laine M . Safety of next-day discharge after transfemoral transcatheter aortic valve replacement with a self-expandable versus balloon-expandable valve prosthesis. Circ Cardiovasc Interv. 2019;12:e007756. 10.1161/CIRCINTERVENTIONS.118.007756 31167602

[18] Nelson PR Kracjer Z Kansal N , et al. A multicenter, randomized, controlled trial of totally percutaneous access versus open femoral exposure for endovascular aortic aneurysm repair (the PEVAR trial). J Vasc Surg. 2014;59:1181–1193. 24440678 10.1016/j.jvs.2013.10.101

[19] Okuyama K Jilaihawi H Kashif M , et al. Transfemoral access assessment for transcatheter aortic valve replacement: evidence-based application of computed tomography over invasive angiography. Circ Cardiovasc Imaging. 2014;8:e001995. 10.1161/CIRCIMAGING.114.001995 25552490

[20] Hayashida K Lefèvre T Chevalier B , et al. Transfemoral aortic valve implantation new criteria to predict vascular complications. JACC Cardiovasc Interv. 2011;4(8):851–858. 21851897 10.1016/j.jcin.2011.03.019

[21] Li F McDermott MM Li D , et al. The association of lesion eccentricity with plaque morphology and components in the superficial femoral artery: a high-spatial-resolution, multi-contrast weighted CMR study. J Cardiovasc Magn Reson. 2010;12:37. 20591197 10.1186/1532-429X-12-37 PMC2904754

[22] Kappetein AP Head SJ Généreux P , et al. Updated standardized endpoint definitions for transcatheter aortic valve implantation: the valve academic research consortium-2 consensus document. J Am Coll Cardiol. 2012;60:1438–1454. 23036636 10.1016/j.jacc.2012.09.001

[23] van Wiechen MP Ligthart JM Van Mieghem NM . Large-bore vascular closure: new devices and techniques. Interv Cardiol. 2019;14:17–21. 30858887 10.15420/icr.2018.36.1 PMC6406132

[24] Krajcer Z Wood DA Strickman N , et al. Pivotal clinical study to evaluate the safety and effectiveness of the MANTA vascular closure device during percutaneous EVAR and TEVAR procedures. J Endovasc Ther. 2020;27:414–420. 32193971 10.1177/1526602820912224

[25] Honda Y Araki M Yamawaki M , et al. The novel echo-guided ProGlide technique during percutaneous transfemoral transcatheter aortic valve implantation. J Interv Cardiol. 2018;31:216–222. 29193337 10.1111/joic.12468

[26] Lin SY Lyu SY Su TW , et al. Predictive factors for additional proglide deployment in percutaneous endovascular aortic repair. J Vasc Interv Radiol. 2017;28(4):570–575. 28190708 10.1016/j.jvir.2016.12.1219

[27] Manunga JM Gloviczki P Oderich GS , et al. Femoral artery calcification as a determinant of success for percutaneous access for endovascular abdominal aortic aneurysm repair. J Vasc Surg. 2013;58(5):1208–1212. 23830310 10.1016/j.jvs.2013.05.028

[28] Stone MB Moon C Sutijono D , et al. Needle tip visualization during ultrasound-guided vascular access: short-axis vs long-axis approach. Am J Emerg Med. 2010;28(3):343–347. 20223394 10.1016/j.ajem.2008.11.022

[29] Kroon HG Tonino PAL Savontaus M , et al. Dedicated plug based closure for large bore access -The MARVEL prospective registry. Catheter Cardiovasc Interv. 2020; 97:1270–1278. doi:10.1002/ccd.29439. 33347739 PMC8246962

[30] van Wiechen MP Kroon H Hokken TW , et al. Vascular complications with a plug-based vascular closure device after transcatheter aortic valve replacement: predictors and bail-outs. Catheter Cardiovasc Interv. 2021;98(5):e737-e745. doi:10.1002/ccd.29506. PMC9292646 33533544

[31] Montero-Cabezas JM van der Meer RW van der Kley F , et al. Percutaneous decannulation of femoral venoarterial ECMO cannulas using MANTA vascular closure device. Can J Cardiol. 2019;35(6):796.e9–796.e11. 10.1016/j.cjca.2019.02.010 31151721

[32] Dahlbacka S Vähäsilta T Moriyama N , et al. Ultrasound-navigated MANTA™ deployment after removal of extracorporeal membrane oxygenation cannula. Ann Thorac Surg. 2020; 110(4):e307–e309. 10.1016/j.athoracsur.2020.01.064 32145194

